# Serum procalcitonin as an early marker associated with postoperative complications following liver resection: A prospective observational study

**DOI:** 10.1371/journal.pone.0338171

**Published:** 2026-09-23

**Authors:** Mohammad Saydul Aman, Md. Shawkat Ali, Bishow Raj Khadka, Bineet Kumar Yadav, Bidhan Chandra Das, Md Foyjul Islam

**Affiliations:** 1 Department of Surgical Gastroenterology, National Gastroliver Institute and Hospital, Dhaka, Bangladesh; 2 Department of Surgery, Rangpur Medical College Hospital, Rangpur, Bangladesh; 3 Department of General Surgery, Patan Academy of Health Sciences, Lagankhel, Lalitpur, Bagmati, Nepal; 4 Department of General Surgery, Nobel Medical College Teaching Hospital Ltd., Morang, Nepal; 5 Department of Hepatobiliary, Pancreatic, and Liver Transplantation Surgery, Bangladesh Medical University, Dhaka, Bangladesh; 6 Department of Epidemiology, Institute of Epidemiology Disease Control and Research (IEDCR), Dhaka, Bangladesh; Jichi Medical University: Jichi Ika Daigaku, JAPAN

## Abstract

Postoperative complications (POC) following liver resection (LR) can significantly affect patient outcomes, and early predictors are crucial for timely intervention, particularly in resource-limited settings such as Bangladesh. In this prospective observational study conducted from August 2021 to July 2022, 42 patients undergoing LR were enrolled and divided into two groups based on the presence (n = 22) or absence (n = 20) of POC. Serum procalcitonin (PCT), white blood cell count (WBC), and C-reactive protein (CRP) were measured on the first, third, and seventh postoperative days (POD). Median serum PCT levels were significantly higher in patients with POC on all PODs (POD1: 1.71 vs. 0.53 µg/L, p = 0.001; POD3: 0.69 vs. 0.23 µg/L, p = 0.001; POD7: 0.48 vs. 0.10 µg/L, p = 0.003), whereas WBC and CRP did not differ between groups. The area under the receiver operating characteristic curve (AUC) for serum PCT was 0.798 (95% CI: 0.655–0.927), 0.797 (95% CI: 0.648–0.920), and 0.769 (95% CI: 0.614–0.898) on POD1, POD3, and POD7, respectively, indicating that POD1 serum PCT showed moderate discriminatory ability for the broad composite endpoint. Using the Youden index, the optimal POD1 serum PCT cutoff value was identified as 1.240 µg/L, which achieved 86.4% sensitivity and 70.0% specificity. In exploratory multivariable analysis, higher POD1 serum PCT was associated with postoperative complications after adjustment for prespecified covariates (adjusted OR: 12.30; 95% CI: 1.44–104.89; p = 0.022); however, the wide confidence interval and small number of events warrant cautious interpretation. Sensitivity analyses showed reduced discrimination for Clavien–Dindo grade ≥II (AUC: 0.650) and grade ≥III complications (AUC: 0.581), indicating limited discriminatory ability for severe morbidity in this dataset. These findings suggest that POD1 PCT may support early postoperative risk stratification, but its clinical utility, particularly for severe morbidity, requires validation in larger studies.

## Introduction

Liver resection (LR) is regarded as the gold-standard surgical approach for various liver lesions. Despite being a highly invasive procedure, it has become safer in recent years. Appropriate patient selection, advances in surgical and anesthetic techniques, the use of minimally invasive surgery, and improvements in perioperative care have expanded the indications for elective LR. In the last two decades, these developments have reduced post-operative mortality from more than 10% to less than 5% [1–3]. However, post-operative morbidity remains high, at around 20–45% in various studies [3–5].

Generally, early detection of postoperative complications (POC) after elective LR is a challenge for liver surgeons. Various parameters such as white blood cell (WBC), C-reactive protein (CRP), interleukin-6 (IL-6), international normalized ratio (INR), serum albumin, serum bilirubin, serum creatinine, serum transaminases, etc., are commonly used for early prediction with variable results [6–10]. Most of these either exhibited low sensitivity or low specificity for morbidity and mortality [3]. The commonly used CRP usually peaks on the second or third post-operative day (POD) and reduces gradually afterwards [11]. Most of the other markers are not useful prior to the fifth POD. Moreover, multiple studies have shown that peri-operative steroids can cause a significant reduction of some markers (CRP, IL-6) in the early post-operative period [11,12]. As a result, it has become difficult to use these markers for prediction.

Procalcitonin (PCT), the 116-amino-acid precursor of calcitonin, is generally produced by thyroid C-cells in healthy people [13]. Various types of stimuli, such as surgery and trauma, alter this typical source of PCT synthesis. Many extra-thyroid tissues (e.g., liver, testis, lung, kidney, etc.) highly express the calcitonin gene CALC-1 [14]. Also, hepatic macrophages have very high expression of PCT-mRNA. Research has proven that acute hepatocyte injury upregulates PCT synthesis in the liver [15,16]. So, after surgery, the PCT concentration may increase 100-fold to 10,000-fold from a baseline of 0.5 µg/L. Steroid administration has no effect on its production. Serum PCT has a 25–30 hour half-life and can reach peak levels within 18–24 hours post-surgery [10,16,17]. A drop in PCT level (compared to the previous day’s value) indicates that the ongoing treatment is effective, and the prognosis is favorable. On the other hand, a persistently raised value over time is associated with a worse prognosis and may indicate an impending complication.

Safe liver resection is paramount for a safe outcome. In low-income countries like Bangladesh, where resources are limited and data regarding elective LR is scarce, an early predictor is essential to ensure a safe outcome. Recently, serum PCT is being measured in the early postoperative period after many major surgeries in resource-rich settings in developed countries as a better prognostic indicator and therapeutic guide [10,11,18]. Some studies suggest that measuring serum PCT between 12 and 48 hours after elective LR could help figure out an adverse outcome, like post-hepatectomy liver failure (PHLF) or death [19,20]. However, there are no such studies on the South Asian cohort that show how serum PCT changes after elective LR. Its impact on clinical practice requires proper evaluation by a prospective study.

This prospective observational study aimed to identify the role of serum PCT levels in the early prediction of POC after elective LR in comparison to common markers (WBC and CRP).

## Materials and methods

### Study design

This prospective observational study was conducted from August 2021 to July 2022 in the Department of Hepatobiliary, Pancreatic, and Liver Transplant Surgery of the Bangladesh Medical University, Dhaka. Patients were enrolled based on the following inclusion criteria: (a) patients undergoing elective LR; (b) age ≥ 18 years; (c) any gender; and (d) Child-Pugh A or B. The exclusion criteria were: (1) LR following trauma; (2) Child-Pugh C; (3) any unplanned LR; (4) patients without written informed consent; and (5) patients with incomplete post-operative data.

### Data collection, surgical procedure, operational definitions, and clinical management

The data collection of study participants was conducted from 05/08/2021 to 30/07/2022. Considering the enrolment criteria, forty-two consecutive patients were enrolled in this study. All of them were evaluated preoperatively through proper history-taking, physical examination, laboratory investigations, and imaging studies. Before every surgery, a clinical discussion meeting was conducted to ensure that LR was well planned. The general condition of all patients was optimized to improve their performance status [21]. All patients were evaluated pre-operatively by anesthesiologists using the American Society of Anesthesiologists score [22].

All patients underwent LR under general anesthesia with tracheal intubation. They were operated on through an open approach (right subcostal or reverse L incision). After a thorough assessment of the peritoneal cavity, attention was given to hepatic inflow and outflow control. Parenchymal transection was done either by the clamp-crush technique or by using an ultrasonic dissector, depending on the surgeon’s choice. If required, concomitant procedures were also performed based on indications (e.g., biliary bypass for recurrent hepatolithiasis, radical lymphadenectomy for gall bladder malignancy, etc.). Special attention was given to proper hemostasis and the prevention of bile leakage. All the resected specimens were sent for histopathology. “The Brisbane 2000 terminology of liver anatomy and resections” was used to define all procedures, e.g., name, type, extent, order, etc., for uniform documentation [23,24]. Major resection was defined as a resection of three or more contiguous or non-contiguous liver segments [2,7]. After recovery, all patients were kept in the post-operative ward overnight and shifted to their respective wards the next morning. No patients received perioperative steroids.

Within the next 30 days, if any POC happened, it was noted and dealt with according to standard hospital protocol. Standard operational definitions of the International Study Group of Liver Surgery were used for defining PHLF [25], post-hepatectomy hemorrhage (PHH) [26], and bile leak [27]. Other complications were also defined according to the standard protocol.

Following hospital discharge, all patients were seen in the outpatient department within the next 30 days.

### PCT measurement

Serum samples were collected in the mornings of the 1st, 3rd, and 7th POD from a peripheral vein and sent for WBC count, CRP, and PCT measurement. The PCT assay was done in the Department of Biochemistry & Molecular Biology of the same hospital and the Siemens Atellica IM BRAHMS Procalcitonin (PCT) analyzer (reference 11202699) was used with a measuring interval of 0.03–50.00 µg/L. No postoperative PCT, WBC, or CRP measurements were missing. Very-low values recorded below the nominal lower measuring interval in the database were retained as recorded; no value exceeded the upper interval.

WBC count and CRP concentration were generally used as predictive markers after surgery in our hospital. By contrast, because no clear criteria for the PCT concentration were available during the study period, the PCT level did not affect the clinical treatment of the patients. Based on the patient’s clinical status, if necessary, any other hematological, biochemical, or imaging investigations were done and repeated.

### Grouping

After surgery, patients were categorized into two groups based on the occurrence of postoperative complications (POC): the “No POC” group, which included patients who did not develop postoperative complications, and the “POC” group, which included patients who developed one or more postoperative complications. The relationship between postoperative inflammatory markers (WBC count, CRP, and PCT) and the occurrence of POC was subsequently evaluated.

### Statistical analysis

Statistical analyses were carried out using the Statistical Package for Social Sciences (SPSS) version 25.0 by SPSS Inc. (IBM), New York, USA. Continuous variables were assessed for normality using the Shapiro-Wilk test and visual inspection. Approximately symmetric variables are presented as mean ± standard deviation and were compared using the independent-samples t test. Skewed variables are presented as median (interquartile range) and were compared using the Mann-Whitney U test. Categorical variables are presented as number (percentage) and were compared using the chi-square test or Fisher’s exact test, as appropriate. Receiver operating characteristic curves were used to assess the discriminatory ability of postoperative PCT, and the optimal exploratory cut-off was selected using the Youden index. We report area under the curve values with 95% confidence intervals and sensitivity/specificity with 95% confidence intervals. An exploratory multivariable logistic regression model was fitted for any postoperative complication using POD 1 PCT and a limited set of prespecified clinical covariates because of the small sample size. p < 0.05 was considered statistically significant.

### Ethical statement

The study was approved by the Institutional Review Board (IRB) of Bangladesh Medical University (BMU), Dhaka (Registration No. 3530; approval granted on 15 July 2021). The research was conducted in accordance with the Declaration of Helsinki. Written informed consent was obtained from all participants prior to enrollment, and confidentiality of patient data was strictly maintained. All patients received standard postoperative care, and study-related investigations did not influence clinical management.

## Results

A total of 42 patients undergoing LR were included in the analysis; 20 patients had no postoperative complications (No POC group), whereas 22 patients developed at least one postoperative complication within 30 days after surgery (POC group). In both groups, patients’ mean age, mean body mass index, performance status, ASA score, and Child-Pugh score were comparable. Regarding gender, male patients were more associated with POC than female patients in our study (68.2% vs. 31.8%, p < 0.05). Except for diabetes, other comorbidities had no statistically significant association with POC. The median MELD score was significantly higher in those having POC (6.00 vs. 7.00, p < 0.05). However, other pre-operative hematological and biochemical functions were comparable between the two groups (Table 1).

**Table 1:** Baseline patient characteristics according to postoperative complication status (N = 42).

Variable	Category	Total (N = 42)	No POC (n = 20)	POC (n = 22)	p-value
Age, years		47.00 ± 4.02	46.95 ± 13.05	47.05 ± 15.15	0.983
Sex	Male	21 (50.0)	6 (30.0)	15 (68.2)	0.029
	Female	21 (50.0)	14 (70.0)	7 (31.8)	
BMI, kg/m^2^		22.16 ± 3.79	22.31 ± 4.09	22.02 ± 3.60	0.808
WHO performance status	0	30 (71.4)	16 (80.0)	14 (63.6)	0.206
	1	9 (21.4)	4 (20.0)	5 (22.7)	
	2	3 (7.1)	0 (0.0)	3 (13.6)	
ASA score	I	22 (52.4)	8 (40.0)	14 (63.6)	0.091
	II	16 (38.1)	11 (55.0)	5 (22.7)	
	III	4 (9.5)	1 (5.0)	3 (13.6)	
Child-Pugh score	A	39 (92.9)	19 (95.0)	20 (90.9)	1.000
	B	3 (7.1)	1 (5.0)	2 (9.1)	
MELD score		7.00 (6.00–8.00)	6.00 (6.00–7.00)	7.00 (7.00–8.75)	0.006
Comorbidities	No	12 (28.6)	4 (20.0)	8 (36.4)	0.315
	Yes	30 (71.4)	16 (80.0)	14 (63.6)	
Hypertension	No	29 (69.0)	12 (60.0)	17 (77.3)	0.320
	Yes	13 (31.0)	8 (40.0)	5 (22.7)	
Diabetes	No	30 (71.4)	11 (55.0)	19 (86.4)	0.040
	Yes	12 (28.6)	9 (45.0)	3 (13.6)	
Previous upper abdominal surgery	No	32 (76.2)	15 (75.0)	17 (77.3)	1.000
	Yes	10 (23.8)	5 (25.0)	5 (22.7)	
Respiratory disease (e.g., COPD, asthma)	No	36 (85.7)	19 (95.0)	17 (77.3)	0.187
	Yes	6 (14.3)	1 (5.0)	5 (22.7)	
Ischemic heart disease	No	38 (90.5)	18 (90.0)	20 (90.9)	1.000
	Yes	4 (9.5)	2 (10.0)	2 (9.1)	
Chronic kidney disease	No	39 (92.9)	19 (95.0)	20 (90.9)	1.000
	Yes	3 (7.1)	1 (5.0)	2 (9.1)	
Hemoglobin, g/dL		12.26 ± 1.80	11.84 ± 1.31	12.64 ± 2.11	0.144
Hematocrit, %		37.46 ± 4.74	36.67 ± 3.51	38.17 ± 5.63	0.302
Red blood cells, × 10^12^/L		4.51 ± 0.59	4.51 ± 0.51	4.51 ± 0.68	1.000
WBC, × 10^9^/L		8.14 ± 2.09	8.36 ± 2.03	7.94 ± 2.17	0.519
Platelet, × 10^9^/L		288.62 ± 101.96	318.65 ± 89.15	261.32 ± 107.08	0.066
Serum total bilirubin, mg/dL		1.09 ± 2.46	0.56 ± 0.25	1.57 ± 3.35	0.171
Serum albumin, g/L		41.06 ± 5.51	40.90 ± 4.44	41.21 ± 6.43	0.856
INR		1.00 ± 0.15	0.97 ± 0.14	1.02 ± 0.17	0.275
ALT, U/L		37.90 ± 22.64	31.80 ± 22.71	43.45 ± 21.60	0.097
Serum creatinine, mg/dL		0.93 ± 0.31	0.86 ± 0.19	1.00 ± 0.38	0.136
eGFR, mL/min/1.73 m^2^		90.45 ± 28.50	88.55 ± 23.99	92.18 ± 32.53	0.681

Data are *n* (%), mean ± SD, or median (IQR). MELD presented as median (IQR) due to skewness; BMI, body mass index; WHO, World Health Organisation; ASA, American Society of Anesthesiologists; MELD, model for end-stage liver disease; COPD, chronic obstructive pulmonary disease; IHD, ischemic heart disease; CKD, chronic kidney disease; WBC, white blood cells; INR, international normalized ratio; ALT, alanine aminotransferase; eGFR, estimated glomerular filtration rate.

In our study, there were 26 benign cases and 16 malignant cases for which elective LR was performed. Among benign cases, the most common indication was bilateral hepatolithiasis and symptomatic giant hepatic hemangioma (seven cases each). All patients with bilateral hepatolithiasis were faced with a POC after surgery (p < 0.05). Among the malignant cases, the most common indication of LR was hepatocellular carcinoma and gall bladder mass (eight cases each) (Table 2).

**Table 2:** Indications of Liver Resection.

Indications	Total (N = 42)		No POC (n = 20)		POC (n = 22)		p-value
**Benign indications**, n (%)	26	(61.9)	12	(60.0)	14	(63.6)	
• Hepatolithiasis (Bilateral)	7	(16.7)	0	(0.0)	7	(31.8)	**0.009**
• Giant hepatic hemangioma	7	(16.7)	4	(20.0)	3	(13.6)	0.691
• Hepatolithiasis (Left)	5	(11.9)	4	(20.0)	1	(4.5)	0.174
• Hepatic adenoma	2	(4.8)	1	(5.0)	1	(4.5)	1.000
• Biliary stricture	2	(4.8)	1	(5.0)	1	(4.5)	1.000
• Mucinous cystic neoplasm	1	(2.4)	1	(5.0)	0	(0.0)	0.476
• Intrahepatic choledochal cyst	1	(2.4)	1	(5.0)	0	(0.0)	0.476
• Simple hepatic cyst	1	(2.4)	0	(0.0)	1	(4.5)	1.000
**Malignant indications**, n (%)	16	(38.1)	8	(40.0)	8	(36.4)	
• Hepatocellular carcinoma	8	(19.0)	3	(15.0)	5	(22.7)	0.700
• Gall bladder mass	8	(19.0)	5	(25.0)	3	(13.6)	0.445

Data are presented as n (%).

In our study, minor resections were the most frequently performed procedures (78.6%), with comparable distributions between the No POC and the POC groups (80.0% vs. 77.3%, respectively; p = 0.830). Anatomical resections accounted for 71.4% of all procedures and did not differ significantly between groups (65.0% vs. 77.3%; p = 0.379). The most commonly performed operations were hepatic bisegmentectomy (38.1%) and left lateral sectionectomy (33.3%), with no significant association between the type of operation and postoperative complications (p = 0.650). Similarly, the order of liver resection (first-, second-, or third-order resection) was comparable between groups (p = 0.810). Pringle maneuver was used in 33.3% of patients overall and showed no significant association with postoperative complications (30.0% vs. 36.4%; p = 0.662). Likewise, the method of parenchymal transection (clamp-crush versus ultrasonic dissector) was not significantly associated with postoperative complications (p = 0.746). However, patients in whom concomitant surgical procedures were done were seen significantly more often among the POC group than the No POC group (81.8% vs. 35.0%; p = 0.002). Median operative time was longer in the POC group than in the No POC group [217.50 (IQR: 153.75–281.25) vs. 187.50 (IQR: 150.00–247.50) minutes], although the difference was not statistically significant (p = 0.614). The POC group had significantly greater intraoperative blood loss compared with the No POC group [400.00 (IQR: 300.00–600.00) mL vs. 300.00 (IQR: 200.00–300.00) mL, p = 0.041]. Furthermore, postoperative hospital stay was significantly longer in the POC group than in the No POC group [13.00 (IQR: 9.00–17.00) days vs. 9.50 (IQR: 7.00–12.25) days, p = 0.013] (Table 3).

**Table 3:** Distribution of study participant by Operative procedures (N = 42).

Variable	Total (N = 42)	No POC (n = 20)	POC (n = 22)	p-value
**Extent of resection**				
Minor (<3 segments)	33 (78.6)	16 (80.0)	17 (77.3)	**0.830**
Major (≥3 segments)	9 (21.4)	4 (20.0)	5 (22.7)	
**Type of resection**				
Anatomical	30 (71.4)	13 (65.0)	17 (77.3)	**0.379**
Non-anatomical	12 (28.6)	7 (35.0)	5 (22.7)	
**Name of operation**				
Hepatic bisegmentectomy^a^	16 (38.1)	8 (40.0)	8 (36.4)	**0.650**
Left lateral sectionectomy (i.e., bisegmentectomy 2, 3)	14 (33.3)	6 (30.0)	8 (36.4)	
Left hepatectomy	6 (14.3)	3 (15.0)	3 (13.6)	
Right hepatectomy	2 (4.8)	1 (5.0)	1 (4.5)	
Right posterior sectionectomy	2 (4.8)	2 (10.0)	0 (0.0)	
Segmentectomy 6	1 (2.4)	0 (0.0)	1 (4.5)	
Left lateral sectionectomy + segmentectomy 6	1 (2.4)	0 (0.0)	1 (4.5)	
**Order of resection**				
First-order	8 (19.0)	4 (20.0)	4 (18.2)	**0.810**
Second-order	16 (38.1)	8 (40.0)	8 (36.4)	
Third-order	17 (40.5)	8 (40.0)	9 (40.9)	
Extended	1 (2.4)	0 (0.0)	1 (4.5)	
**Inflow occlusion (Pringle)**				
No	28 (66.7)	14 (70.0)	14 (63.6)	**0.662**
Yes	14 (33.3)	6 (30.0)	8 (36.4)	
**Transection technique**				
Clamp-crush	20 (47.6)	9 (45.0)	11 (50.0)	**0.746**
Ultrasonic dissector	22 (52.4)	11 (55.0)	11 (50.0)	
**Concomitant intra-abdominal procedure**				
No	17 (40.5)	13 (65.0)	4 (18.2)	**0.002**
Yes	25 (59.5)	7 (35.0)	18 (81.8)	
**Operative time, minutes**	207.50 (150.00–270.00)	187.50 (150.00–247.50)	217.50 (153.75–281.25)	**0.614**
**Amount of blood loss, mL**	300.00 (250.00–500.00)	300.00 (200.00–300.00)	400.00 (300.00–600.00)	**0.041**
**Pringle maneuver**				
No	28 (66.7)	14 (70.0)	14 (63.6)	**0.750**
Yes	14 (33.3)	6 (30.0)	8 (36.4)	
**Parenchymal transection technique**				
Clamp-crush	20 (47.6)	9 (45.0)	11 (50.0)	**0.767**
Ultrasonic dissector/CUSA	22 (52.4)	11 (55.0)	11 (50.0)	
**Postoperative hospital stay, days**	12.00 (8.00–15.50)	9.50 (7.00–12.25)	13.00 (9.00–17.00)	**0.013**

Data are presented as n (%) or mean ± standard deviation (SD). Skewed continuous variables are expressed as median (IQR) and were compared using the Mann–Whitney U test. P values were derived using Fisher’s exact test for 2 × 2 tables and the chi-square test for larger contingency tables. Hepatic bisegmentectomy included bisegmentectomy segments 4b and 5, and segments 5 and 6. *Concomitant intra-abdominal procedures included biliary bypass, radical lymph node dissection, and related procedures.

**Fig 1:**
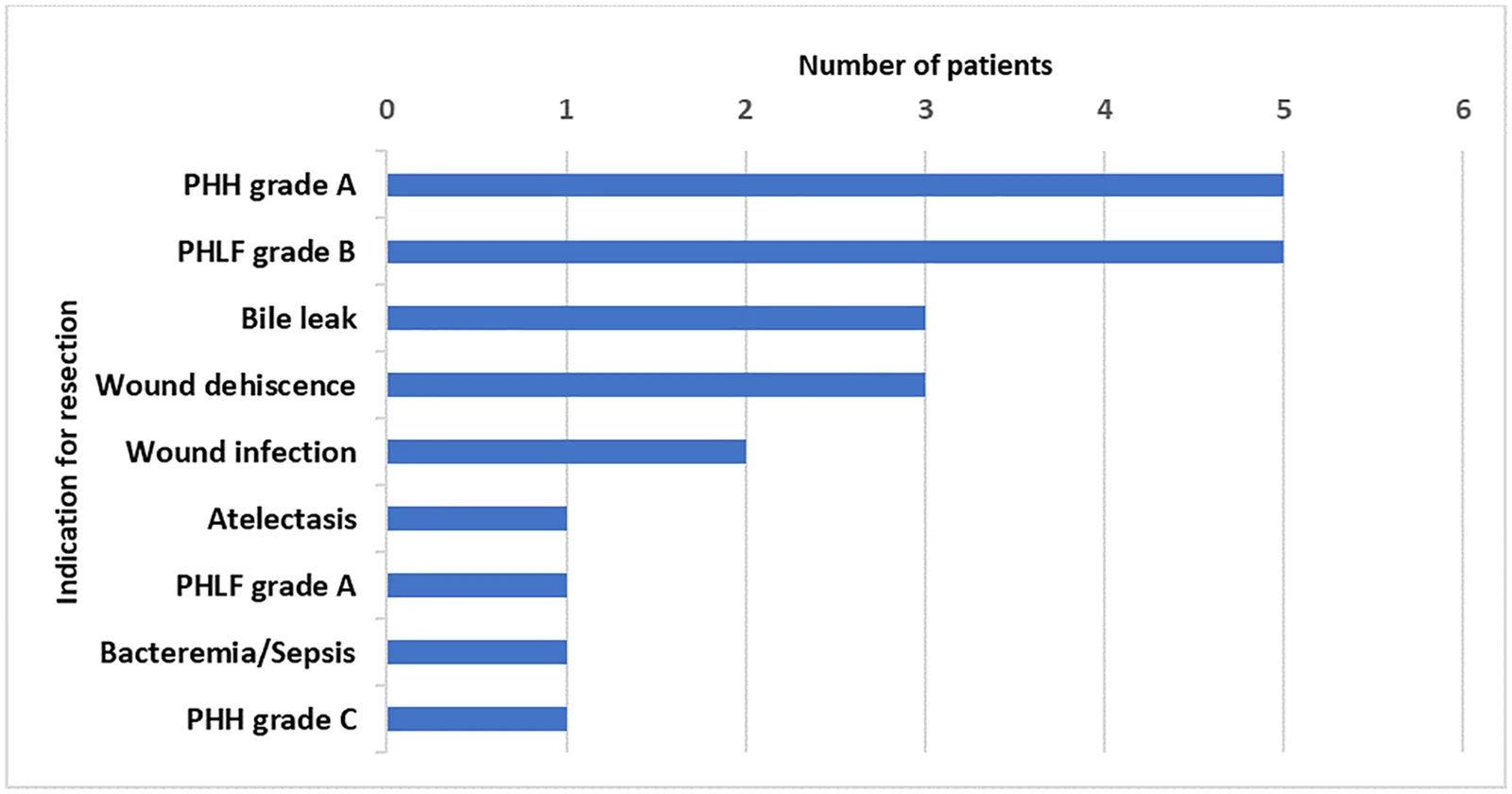
Distribution of study participant by Post-Operative Complications (POC). n (22).

Among the POC, the most common complications were PHH grade A (five cases, 11.9%) and PHLF grade B (five cases, 11.9%). Three cases (7.1%) had bile leakage, three cases (7.1%) had wound dehiscence, and two cases (4.8%) had wound infection (Fig 1). In this one-year LR series, there were no 30-day mortalities.

**Fig 2:**
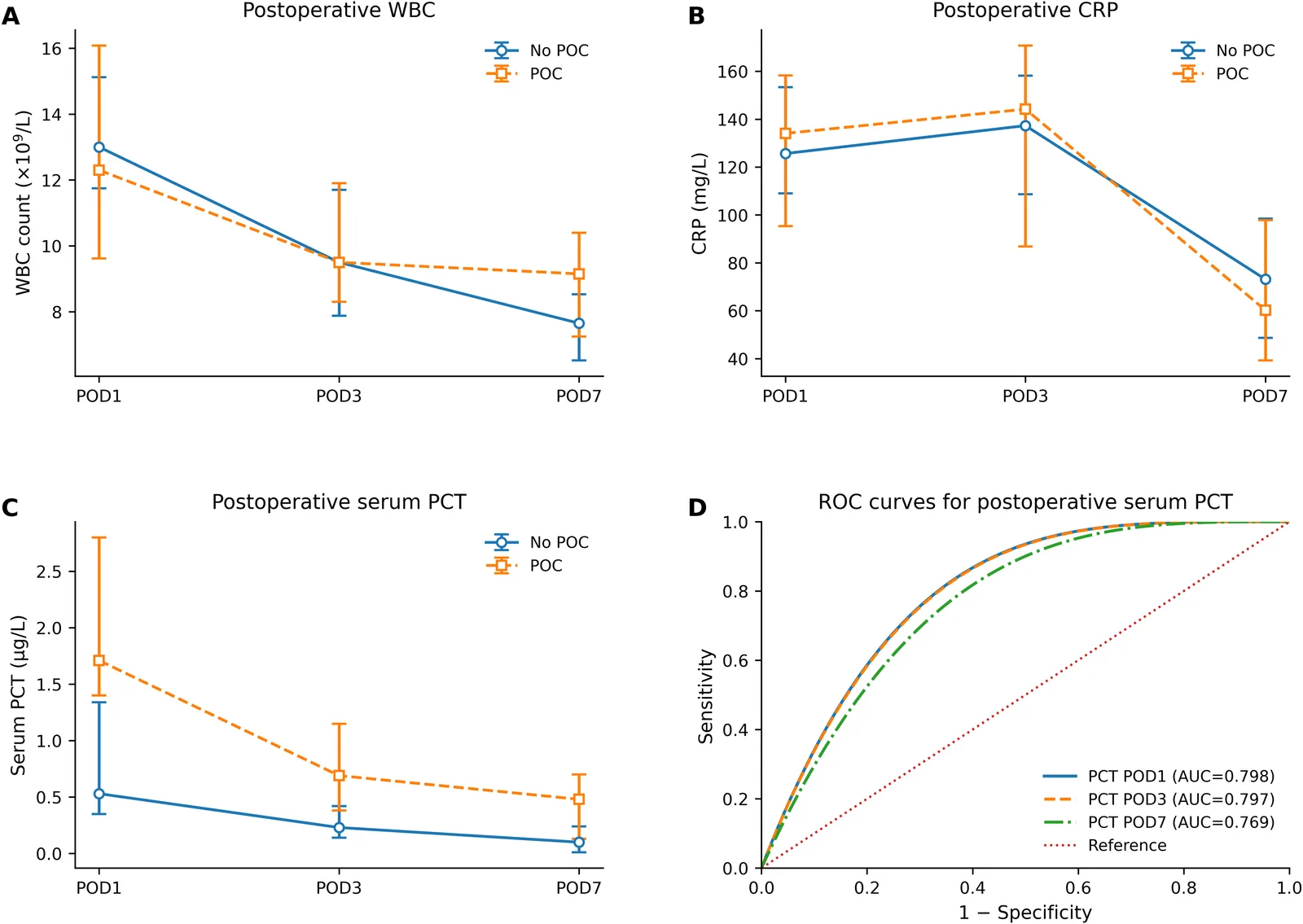
Serial postoperative inflammatory markers and ROC curves. (A) Median WBC count by complication group. (B) Median CRP level by complication group. (C) Median serum PCT level by complication group. (D) Receiver operating characteristic (ROC) curves for serum PCT on POD1 (blue line), POD3 (red line), and POD7 (green line) for discrimination of the broad composite postoperative complication endpoint after elective liver resection. The diagonal line (orange) indicates the reference line. Diagonal line (orange) indicates the reference line.

There were no statistically significant differences in median WBC counts between the No POC and the POC groups on POD 1, POD 3, or POD 7 (p = 0.623, 0.980, and 0.076, respectively) (Fig 2A). Similarly, median CRP levels did not differ significantly between groups at any postoperative time point (p = 0.930, 0.715, and 0.811 for POD 1, POD 3, and POD 7, respectively) (Fig 2B). In contrast, median serum PCT levels were significantly higher among patients who developed postoperative complications compared with those without complications across all postoperative time points. On POD 1, median serum PCT was 1.71 µg/L (IQR: 1.40–2.80) in the POC group versus 0.53 µg/L (IQR: 0.35–1.34) in the No POC group (p = 0.001). Similar significant differences were observed on POD 3 [0.69 µg/L (IQR: 0.38–1.15) vs. 0.23 µg/L (IQR: 0.14–0.42), p = 0.001] and POD 7 [0.48 µg/L (IQR: 0.13–0.70) vs. 0.10 µg/L (IQR: 0.01–0.24), p = 0.003] (Fig 2C; Table 4).

**Table 4:** Comparison of postoperative WBC count, CRP, and serum PCT between patients with and without postoperative complications (N = 42).

Variable	Total (N = 42)	No POC (n = 20)	POC (n = 22)	p-value
**WBC POD1, × 10⁹/L**	12.62 (10.53–15.66)	13.00 (11.75–15.12)	12.30 (9.62–16.08)	0.623
**WBC POD3, × 10⁹/L**	9.50 (8.06–11.90)	9.50 (7.88–11.70)	9.50 (8.30–11.90)	0.980
**WBC POD7, × 10⁹/L**	8.10 (6.78–10.07)	7.65 (6.52–8.53)	9.15 (7.25–10.40)	0.076
**CRP POD1, mg/L**	128.80 (100.75–154.72)	125.65 (109.05–153.38)	134.05 (95.35–158.29)	0.930
**CRP POD3, mg/L**	144.18 (98.88–163.30)	137.30 (108.60–158.15)	144.18 (86.85–170.73)	0.715
**CRP POD7, mg/L**	65.46 (39.21–98.39)	73.12 (48.62–98.45)	60.23 (39.21–97.85)	0.811
**PCT POD1, µg/L**	1.41 (0.48–2.12)	0.53 (0.35–1.34)	1.71 (1.40–2.80)	0.001
**PCT POD3, µg/L**	0.41 (0.18–0.81)	0.23 (0.14–0.42)	0.69 (0.38–1.15)	0.001
**PCT POD7, µg/L**	0.21 (0.06–0.56)	0.10 (0.01–0.24)	0.48 (0.13–0.70)	0.003

Data are median (IQR). P-values were obtained using the Mann-Whitney U tests.; WBC, white blood cells; POD, post-operative day; CRP, c-reactive protein; PCT, procalcitonin.

Receiver operating characteristic (ROC) curves were constructed to assess the discriminatory ability of serum PCT measured on POD1, POD3, and POD7 for the occurrence of the broad composite postoperative complication endpoint (Fig 2D).

The AUC for POD 1 serum PCT was 0.798 (95% CI: 0.655–0.927), compared with 0.797 (95% CI: 0.648–0.920) for POD 3 and 0.769 (95% CI: 0.614–0.898) for POD 7. Using the Youden index, the optimal cutoff value for POD 1 serum PCT was identified as 1.240 µg/L. At this threshold, the sensitivity was 86.4% (95% CI: 66.7–95.3%) and the specificity was 70.0% (95% CI: 48.1–85.5%). The corresponding positive predictive value (PPV) and negative predictive value (NPV) were 76.0% and 82.4%, respectively. For POD 3 serum PCT, the optimal cutoff value was 0.330 µg/L, yielding a sensitivity of 81.8% and specificity of 65.0%. On POD 7, the optimal cutoff value was 0.315 µg/L, with lower sensitivity (63.6%) but higher specificity (80.0%) (Table 5).

**Table 5:** Area under the curve (AUC) based on the mean serum PCT levels measured on POD 1, 3 and 7.

Variable	AUC	95% CI for AUC	Optimal cutoff (µg/L)*	Sensitivity	95% CI	Specificity	95% CI	PPV	NPV
PCT POD1	0.798	0.655–0.927	1.240	86.4%	66.7–95.3%	70.0%	48.1–85.5%	76.0%	82.4%
PCT POD3	0.797	0.648–0.920	0.330	81.8%	61.5–92.7%	65.0%	43.3–81.9%	72.0%	76.5%
PCT POD7	0.769	0.614–0.898	0.315	63.6%	43.0–80.3%	80.0%	58.4–91.9%	77.8%	66.7%

*Optimal cutoff values were determined using the Youden index. AUC, area under the curve; POD, post-operative day; PCT, procalcitonin; CI, confidence interval; PPV, positive predictive value; NPV, negative predictive value.

In exploratory multivariable logistic regression analysis, higher POD1 serum PCT was associated with postoperative complications after adjustment for sex, preoperative MELD score, and concomitant intra-abdominal procedures (adjusted OR: 12.30, 95% CI: 1.44–104.89; p = 0.022). Given the small number of events and the very wide confidence intervals, these adjusted estimates should be regarded as exploratory rather than evidence of independent prediction. Male sex and concomitant intra-abdominal procedures also showed associations with postoperative complications in the adjusted model, but these estimates were similar imprecise. Using a clinically relevant cutoff, patients with POD1 serum PCT ≥ 1.1 µg/L had significantly higher odds of developing postoperative complications compared with those with lower values (OR: 14.78, 95% CI: 3.14–69.51; p < 0.001). Sensitivity analyses using stricter definitions of clinically relevant complications showed moderate predictive performance of POD1 serum PCT for Clavien–Dindo grade ≥II complications (AUC: 0.650, 95% CI: 0.470–0.812) and lower performance for grade ≥III complications (AUC: 0.581, 95% CI: 0.388–0.768). These findings indicate reduced discrimination when clinically relevant endpoints are considered and limited discriminatory ability for severe postoperative morbidity in the current study (Table 6).

**Table 6:** Additional exploratory analyses of postoperative serum procalcitonin (PCT) for prediction of postoperative complications following liver resection.

Analysis	Variable/ Outcome	Estimate	95% CI	p-value
**Multivariable logistic regression**	POD1 PCT (per 1 µg/L increase)	Adjusted OR: 12.30	1.44–104.89	0.022
	Male sex	Adjusted OR: 33.03	1.54–708.95	0.025
	Preoperative MELD score	Adjusted OR: 2.20	0.80–6.06	0.128
	Concomitant intra-abdominal procedure	Adjusted OR: 51.22	2.48–1058.25	0.011
**Association of POD1 PCT cutoff with postoperative complications**	POD1 PCT ≥ 1.1 µg/L vs. < 1.1 µg/L	OR: 14.78	3.14–69.51	<0.001*
**Sensitivity analysis (ROC performance)**	Clavien–Dindo ≥II complications	AUC: 0.650	0.470–0.812	
	Optimal cutoff	1.240 µg/L		
	Sensitivity	83.3%	60.8–94.2%	
	Specificity	58.3%	38.8–75.5%	
	PPV/ NPV	60.0%/ 82.4%		
	Clavien–Dindo ≥III complications	AUC: 0.581	0.388–0.768	
	Optimal cutoff	1.280 µg/L		
	Sensitivity	87.5%	52.9–97.8%	
	Specificity	50.0%	34.1–65.9%	
	PPV/ NPV	29.2%/ 94.4%		

Multivariable logistic regression model included POD1 serum PCT, sex, preoperative MELD score, and concomitant intra-abdominal procedure. Sensitivity analyses were conducted using stricter definitions of clinically relevant postoperative complications according to Clavien–Dindo classification. * Fisher’s exact test.

Abbreviations: OR, odds ratio; CI, confidence interval; ROC, receiver operating characteristic; AUC, area under the curve; PPV, positive predictive value; NPV, negative predictive value; MELD, model for end-stage liver disease; POD, postoperative day; PCT, procalcitonin.

## Discussion

Liver resection (LR) remains a technically demanding procedure, and although perioperative mortality has declined globally, postoperative morbidity continues to be a major clinical challenge [3]. Evidence from high-income countries shows substantial improvements in perioperative care; however, the applicability of these findings to low-resource settings such as Bangladesh has been insufficiently explored. This prospective study contributes to this gap by evaluating postoperative outcomes following elective LR and, importantly, assessing the utility of serum procalcitonin (PCT) as an early biomarker for postoperative complications (POC).

In this present study serum PCT showed stronger discrimination for the broad composite endpoint than WBC or CRP, but the magnitude of this finding should be interpreted cautiously. PCT levels peaked within the first postoperative day (POD), consistent with findings from Kretzschmar et al., and its rapid induction following hepatocellular injury [17]. POD1 PCT showed moderate discriminatory ability for overall POC (AUC: 0.798; 95% CI: 0.655–0.927), with an exploratory Youden-derived cutoff of 1.240 µg/L yielding 86.4% sensitivity and 70.0% specificity.Higher POD1 PCT was associated with increased odds of postoperative complications in the exploratory adjusted analysis, but the wide confidence interval and limited number of events preclude firm conclusions regarding independent prediction. Accordingly, our results support an association between early PCT elevation and postoperative morbidity rather than establishing PCT as a reliable or specific predictor of all complications. In contrast, WBC count demonstrated nonspecific postoperative fluctuations, and CRP exhibited its characteristic delayed rise, limiting their value for early risk stratification. These findings reaffirm earlier reports indicating the superior early predictive performance of PCT in major abdominal surgery [10,11].

Our resultsbroadly consistent with those of Li et al., who reported an association between PCT ≥ 1.000 µg/L at 12 hours after liver resection and outcomes including PHLF, sepsis, and mortality [20]. However, direct comparison is limited because our primary endpoint was a heterogeneous composite that included both infectious and non-infectious postoperative events. The similarity of thresholds may support the biological responsiveness of PCT after liver resection, but it should not be taken as evidence of equivalent clinical performance across different endpoints or patient populations.Our findings should also be interpreted in light of recent larger-scale evidence. Mori et al. analyzed 301 hepatectomy patients and used an infectious-complication endpoint, reporting that PCT on PODs 1 and 3 was associated with infectious complications after hepatectomy [28]. In contrast, our study prospectively evaluated a much smaller cohort and used a broad composite endpoint incorporating infectious and non-infectious complications. This distinction is important because PCT is biologically more closely related to infection and systemic inflammatory responses than to all forms of postoperative morbidity. The broader endpoint is a feature of the present study but also limits specificity and likely contributes to the uncertainty observed in our estimates.

PCT is usually regarded as a marker of bacterial infection or sepsis. But it also rises as a pro-inflammatory cytokine after major surgery, such as liver resection [10,11,17]. According to Aoki et al. [19], the peak serum PCT within the first two days of surgery can correctly predict Clavien-Dindo complications grade II and above. They set a cutoff value of 0.35 µg/L (AUC, 0.860), with a sensitivity and specificity rate of 80% and 83%, respectively. However, that cutoff value was below the normal baseline level of serum PCT 0.5 µg/L for any clinical application. Using the serum PCT as a predictor of POC necessitates a value greater than 0.5 µg/L. In that sense, it is hard to utilize “0.35 µg/L” as a benchmark for future reference. In our study, the exploratory POD1 cutoff for the broader composite endpoint was 1.240 µg/L, with sensitivity of 86.4% and specificity of 70.0%. Importantly, unlike Aoki et al. [19], we included grade I as well as more severe complications. When we restricted our analysis to clinically relevant outcomes, discriminatory performance decreased to an AUC of 0.650 for Clavien–Dindo grade ≥II and 0.581 for grade ≥III complications. This reduction, particularly for grade ≥III morbidity, indicates that the clinical utility of POD1 PCT for identifying severe complications is limited.

Because PCT is biologically more closely linked to bacterial and systemic inflammatory responses than to all non-infectious postoperative morbidity, our results should not be interpreted as evidence that serum PCT is a specific marker for every postoperative complication. Rather, elevated POD1 PCT was associated with the broad composite of postoperative morbidity in this study and may reflect a mixture of infectious burden, tissue injury, and early adverse postoperative physiology.

We have performed elective LR for 26 benign and 16 malignant diseases. We had no 30-day mortality, though the morbidity rate was slightly on the higher side (52.4%) compared to the literature [2,3]. This may partly reflect careful postoperative surveillance and detailed documentation of even minor complications in our institution.

This study has several limitations. First, the sample size was small (42 patients with 22 composite events) and the study was conducted at a single center, limiting precision and external validity. The exploratory multivariable model therefore had a low event-to-parameter ratio, and the resulting adjusted odds ratios had very wide confidence intervals; these estimates should not be interpreted as definitive evidence of independent prediction. Second, the cohort included a high proportion of benign disease, particularly hepatolithiasis, which differs from many hepatectomy series dominated by malignant indications. Third, preoperative PCT was not measured, preventing assessment of relative postoperative change. Fourth, the primary endpoint was a clinically heterogeneous composite that included both infectious and non-infectious events, although PCT is biologically more closely related to infection and systemic inflammatory responses. Fifth, sensitivity analyses showed lower discriminatory performance for clinically relevant complications, especially Clavien–Dindo grade ≥III events (AUC: 0.581), indicating limited utility for severe morbidity in this dataset. Finally, the adjusted model and cutoff analyses were exploratory and may be affected by overfitting and optimism bias. Larger multicenter studies with prespecified infection-specific and clinically relevant endpoints are needed to validate these findings.

## Conclusion

Serum procalcitonin on postoperative day 1 was associated with broad composite of postoperative complications after elective liver resection and showed moderate discriminatory ability in this prospective cohort. Discrimination decreased when the endpoint was restricted to clinically relevant complications and was low for Clavien–Dindo grade ≥III morbidity. POD1 PCT may therefore have a role in exploratory early postoperative risk stratification, but it should not be considered a specific or reliable predictor of all postoperative complications on the basis of these data. The proposed cutoff and adjusted associations require validation in larger, preferably multicenter studies using prespecified clinically relevant and infection-specific endpoints.

## Supporting information

S1 Dataset De-identified minimal dataset underlying the analyses. (XLS)
