# Supplementary material for: Serum procalcitonin as an early marker associated with postoperative complications following liver resection: A prospective observational study
Source: PLoS One. 2026 Sep 23;21(9):e0338171. doi: 10.1371/journal.pone.0338171 (PMC13600518; doi:10.1371/journal.pone.0338171)
Supplement: Supplementary file 1 [file pone.0338171.s003.docx]

**Response to Reviewers**

Manuscript Title: ***Serum Procalcitonin as an Early Marker Associated with Postoperative Complications Following Liver Resection: A Prospective Observational Study***

Manuscript ID: PONE-D-25-62019

We sincerely thank the Academic Editor and Reviewers for their thoughtful and constructive comments. We have carefully revised the manuscript accordingly. All major concerns were addressed through re-analysis of the dataset, revision of statistical methods, clarification of group definitions, addition of exploratory multivariable analyses, sensitivity analyses, updated ROC analyses with confidence intervals, strengthening of the Discussion, and comprehensive language editing. Revised sections are highlighted in the manuscript.

**Journal Requirements**

**Comment 1**

Please ensure that your manuscript meets PLOS ONE's style requirements.

**Response**

The manuscript has been fully revised according to the PLOS ONE formatting guidelines, including title page structure, table formatting, figure legends, reference style, and manuscript organization. File naming and formatting requirements were also checked and corrected.

**Comment 2**

Funding information and financial disclosure do not match.

**Response**

The Funding Statement and Financial Disclosure sections were revised to ensure consistency across all submission documents. The correct grant information has now been uniformly stated throughout the manuscript and cover letter.

**Comment 3**

Please provide a complete funding statement and include “There was no additional external funding received for this study.”

**Response**

The Funding Statement has been revised accordingly. We now clearly state all sources of support and added the sentence:
“There was no additional external funding received for this study.”

**Comment 4**

Please state the role of the funders.

**Response**

The following statement has been added:
“The funders had no role in study design, data collection and analysis, decision to publish, or preparation of the manuscript.”

**Comment 5**

Please include the full ethics statement.

**Response**

A detailed ethics statement has now been incorporated into the Methods section, including the IRB name, approval number, approval date, and informed written consent procedures.

**Comment 6**

Please upload a clearer version of Figure 2.

**Response**

A high-resolution revised version of Figure 2 with improved labeling and figure quality has been prepared and uploaded.

**Comment 7**

Please anonymize identifying data in the supplementary dataset.

**Response**

The supplementary dataset has been fully de-identified. All potentially identifying variables and hidden metadata were removed prior to re-uploading the revised dataset.

**Comment 8**

Please include captions for supporting information files.

**Response**

Captions for supporting files were added at the end of the manuscript, and in-text citations were updated accordingly.

**Comment 9**

Please evaluate reviewer-suggested references.

**Response**

The recommended study by Mori et al. (Digestive Surgery, 2025) was reviewed, cited, and incorporated into the Introduction and Discussion sections. The discussion was expanded to compare study design, endpoints, patient populations, and predictive performance.

**Reviewer #1**

**Major Comments**

**① Definition of postoperative complications and mismatch with PCT biology**

**Response**

We appreciate this important observation. We agree that PCT is biologically more closely related to infectious complications than all-cause postoperative morbidity. To address this concern:

- We clarified that the primary endpoint was a composite postoperative complication outcome.
- We added a dedicated discussion paragraph acknowledging endpoint heterogeneity.
- We conducted sensitivity analyses using stricter clinically relevant endpoints (Clavien–Dindo ≥II and ≥III complications).
- These additional analyses are now presented in revised Table 8.

The Discussion was revised to explicitly state that elevated PCT may reflect a combination of infectious burden, tissue injury, and adverse postoperative physiology rather than infection alone.

**② Inconsistencies in group definitions and implausible results**

**Response**

We carefully re-checked the dataset, group assignments, statistical analyses, and manuscript text. We identified inconsistencies in earlier labeling of “Group A” and “Group B” and corrected them throughout the manuscript, tables, and figures.

The revised manuscript now consistently defines:

- “No POC group” = patients without postoperative complications
- “POC group” = patients with postoperative complications

All analyses were re-run after verification of correct patient assignment. After correction, clinically expected findings were observed, including:

- Higher blood loss in the POC group
- Longer postoperative hospital stay in the POC group

These corrected results are now presented in revised Tables 4 and corresponding Results text.

**③ Sample size limitations and robustness of ROC analysis**

**Response**

We agree that the small sample size limits precision and robustness. To address this:

- 95% confidence intervals for AUC, sensitivity, and specificity were added.
- The Youden index method for cutoff selection was explicitly described.
- The Discussion and Conclusion were revised to emphasize the exploratory nature of the proposed cutoff.
- Limitations related to optimism bias and overfitting were explicitly acknowledged.

**④ Lack of multivariable analysis and adjustment for confounders**

**Response**

An exploratory multivariable logistic regression analysis was performed using POD1 PCT, sex, MELD score, and concomitant intra-abdominal procedures. POD1 PCT remained independently associated with postoperative complications after adjustment (adjusted OR 12.30; 95% CI 1.44–104.89; p = 0.022). Results are now included in revised Table 8 and Results section. Because of the small sample size, we clearly describe these analyses as exploratory.

**⑤ Statistical methods for continuous variables**

**Response**

Continuous variables were reassessed for normality using the Shapiro–Wilk test and visual inspection. Skewed variables are now presented as median (IQR), and Mann–Whitney U tests were used where appropriate. The Statistical Analysis section was substantially revised to clarify these methods.

**⑥ Positioning within the existing literature**

**Response**

The discussion was expanded substantially to incorporate the recent study by Mori et al. (Digestive Surgery, 2025). We compared:

- infectious vs composite endpoints,
- cohort size,
- patient population,
- statistical precision,
- and predictive performance.

We also clarified how our prospective South Asian cohort differs from larger retrospective studies.

**⑦ Generalizability and patient population**

**Response**

We expanded the Discussion to address:

- the predominance of benign disease,
- hepatolithiasis-related surgery,
- regional surgical patterns,
- and limitations in external validity.

These issues are now acknowledged explicitly in the revised limitations section.

**Minor Comments**

**① Clarity and consistency of tables and figures**

**Response**

All tables and figures were carefully renumbered, cross-checked, and revised for consistency with the manuscript text.

**② Clarification of PCT measurement protocol**

**Response**

The Methods section now clearly specifies:

- exact timing of PCT measurements,
- assay platform used,
- measurement range,
- and handling of missing data.

**③ Definition and grading of complications**

**Response**

Complications are now explicitly described according to standard ISGLS definitions and Clavien–Dindo classification. Additional sensitivity analyses using Clavien–Dindo ≥II and ≥III complications were also added.

**④ Language and style**

**Response**

The manuscript underwent extensive language editing to improve grammar, clarity, consistency, and readability.

**⑤ Ethics and data availability**

**Response**

The ethics approval number and approval date were added. The dataset was anonymized and uploaded as supporting information in accordance with PLOS ONE data-sharing policy.

**Reviewer #2**

**Major Comments**

**1. Confounding and lack of multivariable analysis**

**Response**

An exploratory multivariable logistic regression analysis was performed adjusting for key covariates including sex, MELD score, and concomitant intra-abdominal procedures. POD1 serum PCT remained independently associated with postoperative complications. Results are now presented in revised Table 8.

**2. Definition and heterogeneity of postoperative complications**

**Response**

We agree that the composite endpoint was clinically heterogeneous. We clarified complication definitions, added discussion regarding biological interpretation of PCT, and performed sensitivity analyses for Clavien–Dindo ≥II and ≥III complications. Results are now included in revised Table 8.

**3. Interpretation of ROC analysis and cutoff value**

**Response**

The wording throughout the manuscript was revised to avoid overstating predictive performance. Terms such as “reliable predictor” were replaced with more cautious language such as “moderate discriminatory ability” and “potential early biomarker.” We also explicitly state that the cutoff is exploratory and requires external validation.

**4. Counterintuitive perioperative findings**

**Response**

After rechecking group assignment and re-running analyses, the corrected results now demonstrate higher blood loss and longer postoperative hospital stay among patients with postoperative complications, which is clinically consistent. The Results section and Table 4 were corrected accordingly.

**5. Lack of preoperative PCT measurements**

**Response**

This limitation is now more prominently discussed in the revised limitations paragraph.

**Minor Comments**

**1. Clarify group labeling**

**Response**

All group labels were standardized throughout the manuscript, tables, and figures.

**2. Statistical assumptions**

**Response**

Normality testing procedures and use of non-parametric methods were added to the Statistical Analysis section.

**3. Figures**

**Response**

Figure legends were revised to clarify curve labeling and graphical interpretation. Higher-resolution figures were uploaded.

**4. Language and style**

**Response**

The manuscript underwent comprehensive language revision and editing for clarity and readability.

We again sincerely thank the Academic Editor and Reviewers for their insightful comments, which substantially improved the quality and rigor of our manuscript.
