# Supplementary material for: Serum procalcitonin as an early marker associated with postoperative complications following liver resection: A prospective observational study
Source: PLoS One. 2026 Sep 23;21(9):e0338171. doi: 10.1371/journal.pone.0338171 (PMC13600518; doi:10.1371/journal.pone.0338171)
Supplement: Supplementary file 2 [file pone.0338171.s004.docx]

**Response to Reviewer**

Manuscript ID: **PONE-D-25-62019R1**

Title: **Serum Procalcitonin as an Early Marker Associated with Postoperative Complications Following Liver Resection: A Prospective Observational Study**

Dear Academic Editor and Reviewer,

We sincerely thank you for the careful re-review and constructive comments. We have revised the manuscript to temper causal/predictive language, clarify the exploratory nature of the adjusted analyses, explicitly discuss the reduced discrimination for clinically relevant and severe complications, distinguish our broad composite endpoint from infection-specific endpoints in prior studies, and correct figure/statistical presentation inconsistencies. All changes are highlighted in tracked change manuscript.

# Comment 1: Heterogeneous composite endpoint and overly strong interpretation of PCT.

Response: We agree. We have consistently reframed POD1 PCT as being “associated with” postoperative complications and as showing “moderate discriminatory ability,” rather than as a reliable or specific predictor of all postoperative morbidity. We also explicitly state that the composite endpoint includes infectious and non-infectious events and that PCT may reflect infection, tissue injury, and postoperative inflammatory physiology.

**Changes: Abstract; Discussion paragraphs addressing interpretation and biological specificity; Conclusion.**

# Comment 2: Small sample size, limited events, wide confidence intervals, and wording around “independent predictor.”

Response: We agree. We now explicitly describe the multivariable analysis as exploratory, note the low number of events and very wide confidence intervals, and avoid interpreting the adjusted estimates as definitive evidence of independent prediction. The Results, Discussion, limitations, and Conclusion have been softened accordingly.

**Changes: Abstract; Results paragraph accompanying Table 6; Discussion; Limitations; Conclusion.**

# Comment 3: Sensitivity analyses and limited utility for severe morbidity should be discussed more clearly.

Response: We agree. We now emphasize that discrimination decreased when stricter clinically relevant endpoints were used: AUC 0.650 for Clavien–Dindo grade ≥II and 0.581 for grade ≥III complications. We explicitly acknowledge that the low AUC for grade ≥III morbidity indicates limited clinical discrimination for severe complications in this dataset.

**Changes: Abstract; Results; Discussion; Limitations; Conclusion.**

# Comment 4: Clarify distinction between infection-specific endpoints in previous studies and the broad composite endpoint in the present study.

Response: We agree. We revised the comparison with Li et al., Mori et al., and Aoki et al. to make clear that previous studies often used infection-specific or clinically restricted endpoints, whereas our primary endpoint included both infectious and non-infectious complications. We now present this difference as both a conceptual distinction and a limitation of our study.

**Changes: Discussion paragraphs comparing the present findings with Li et al., Mori et al., and Aoki et al.**

# Comment 5: Figure numbering and inconsistent statistical descriptions.

Response: We carefully reviewed the relevant presentation. The text describing serial WBC, CRP, and PCT now refers to Figure 2A–C, while the ROC analysis refers to Figure 2D; Figure 1 remains the distribution of postoperative complications. We also corrected the Figure 2 legend from “mean” to “median” for the skewed biomarkers and removed the contradictory Table 4 footnote stating that an independent-samples t-test was used. Table 4 now states that the data are median (IQR) and were compared using Mann–Whitney U tests, consistent with the Statistical Analysis section.

**Changes: Results cross-references; Figure 2 legend; Table 4 footnote; Table 5 title.**

We appreciate the reviewer’s comments, which have helped us improve the precision and clarity of the manuscript.

Sincerely,
The Authors
