# Supplementary material for: Serum procalcitonin as an early marker associated with postoperative complications following liver resection: A prospective observational study
Source: PLoS One. 2026 Sep 23;21(9):e0338171. doi: 10.1371/journal.pone.0338171 (PMC13600518; doi:10.1371/journal.pone.0338171)
Supplement: Peer review history

# Peer review: Decision Letter 0

**DOI:** 10.1371/journal.pone.0338171.r001

**Authors:** Taiichi Wakiya

29 Dec 2025

Dear Dr. Islam,

- **Please consider the reviewers' comments in addressing revisions that encompass the included requests for additional information.**

plosone@plos.org. When you're ready to submit your revision, log on to https://www.editorialmanager.com/pone/ and select the 'Submissions Needing Revision' folder to locate your manuscript file.

- A letter that responds to each point raised by the academic editor and reviewer(s). You should upload this letter as a separate file labeled 'Response to Reviewers'.
- A marked-up copy of your manuscript that highlights changes made to the original version. You should upload this as a separate file labeled 'Revised Manuscript with Track Changes'.
- An unmarked version of your revised paper without tracked changes. You should upload this as a separate file labeled 'Manuscript'.

If you would like to make changes to your financial disclosure, please include your updated statement in your cover letter. Guidelines for resubmitting your figure files are available below the reviewer comments at the end of this letter.

If applicable, we recommend that you deposit your laboratory protocols in protocols.io to enhance the reproducibility of your results. Protocols.io assigns your protocol its own identifier (DOI) so that it can be cited independently in the future. For instructions see: https://journals.plos.org/plosone/s/submission-guidelines#loc-laboratory-protocols. Additionally, PLOS ONE offers an option for publishing peer-reviewed Lab Protocol articles, which describe protocols hosted on protocols.io. Read more information on sharing protocols at https://plos.org/protocols?utm_medium=editorial-email&utm_source=authorletters&utm_campaign=protocols.

We look forward to receiving your revised manuscript.

Kind regards,

Taiichi Wakiya

Academic Editor

PLOS One

**Journal Requirements:**

1. When submitting your revision, we need you to address these additional requirements.

Please ensure that your manuscript meets PLOS ONE's style requirements, including those for file naming. The PLOS ONE style templates can be found at

https://journals.plos.org/plosone/s/file?id=wjVg/PLOSOne_formatting_sample_main_body.pdf and

https://journals.plos.org/plosone/s/file?id=ba62/PLOSOne_formatting_sample_title_authors_affiliations.pdf

2. We note that the grant information you provided in the ‘Funding Information’ and ‘Financial Disclosure’ sections do not match.

When you resubmit, please ensure that you provide the correct grant numbers for the awards you received for your study in the ‘Funding Information’ section.

3. Thank you for stating in your Funding Statement:

This study was partially supported by the Research and Development wing of the Bangabandhu Sheikh Mujib Medical University (grant number ANA 5458289).

Please provide an amended statement that declares *all* the funding or sources of support (whether external or internal to your organization) received during this study, as detailed online in our guide for authors at http://journals.plos.org/plosone/s/submit-now.  Please also include the statement “There was no additional external funding received for this study.” in your updated Funding Statement.

Please include your amended Funding Statement within your cover letter. We will change the online submission form on your behalf.

4. Thank you for stating the following financial disclosure:

This study was partially supported by the Research and Development wing of the Bangabandhu Sheikh Mujib Medical University (grant number ANA 5458289).

Please state what role the funders took in the study.  If the funders had no role, please state: "The funders had no role in study design, data collection and analysis, decision to publish, or preparation of the manuscript."

If this statement is not correct you must amend it as needed.

Please include this amended Role of Funder statement in your cover letter; we will change the online submission form on your behalf.

5. Please include your full ethics statement in the ‘Methods’ section of your manuscript file. In your statement, please include the full name of the IRB or ethics committee who approved or waived your study, as well as whether or not you obtained informed written or verbal consent. If consent was waived for your study, please include this information in your statement as well.

6. Please upload a new copy of Figure 2 as the detail is not clear. Please follow the link for more information:  https://journals.plos.org/plosone/s/figures

7. We note that there is identifying data in the Supporting Information file. Due to the inclusion of these potentially identifying data, we have removed this file from your file inventory. Prior to sharing human research participant data, authors should consult with an ethics committee to ensure data are shared in accordance with participant consent and all applicable local laws.

Data sharing should never compromise participant privacy. It is therefore not appropriate to publicly share personally identifiable data on human research participants. The following are examples of data that should not be shared:

-Name, initials, physical address

-Ages more specific than whole numbers

-Internet protocol (IP) address

-Specific dates (birth dates, death dates, examination dates, etc.)

-Contact information such as phone number or email address

-Location data

-ID numbers that seem specific (long numbers, include initials, titled “Hospital ID”) rather than random (small numbers in numerical order)

Data that are not directly identifying may also be inappropriate to share, as in combination they can become identifying. For example, data collected from a small group of participants, vulnerable populations, or private groups should not be shared if they involve indirect identifiers (such as sex, ethnicity, location, etc.) that may risk the identification of study participants.

Additional guidance on preparing raw data for publication can be found in our Data Policy (https://journals.plos.org/plosone/s/data-availability#loc-human-research-participant-data-and-other-sensitive-data) and in the following article: http://www.bmj.com/content/340/bmj.c181.long.

Please remove or anonymize all personal information (<specific identifying information in file to be removed>), ensure that the data shared are in accordance with participant consent, and re-upload a fully anonymized data set. Please note that spreadsheet columns with personal information must be removed and not hidden as all hidden columns will appear in the published file.

8. Please include captions for your Supporting Information files at the end of your manuscript, and update any in-text citations to match accordingly. Please see our Supporting Information guidelines for more information: http://journals.plos.org/plosone/s/supporting-information.

9. If the reviewer comments include a recommendation to cite specific previously published works, please review and evaluate these publications to determine whether they are relevant and should be cited. There is no requirement to cite these works unless the editor has indicated otherwise. 

[Note: HTML markup is below. Please do not edit.]

Reviewers' comments:

**Comments to the Author**

1. Is the manuscript technically sound, and do the data support the conclusions?

Reviewer #1: Yes

Reviewer #2: Partly

**********

2. Has the statistical analysis been performed appropriately and rigorously?

Reviewer #1: Yes

Reviewer #2: No

**********

3. Have the authors made all data underlying the findings in their manuscript fully available?

The PLOS Data policy

Reviewer #1: No

Reviewer #2: Yes

**********

4. Is the manuscript presented in an intelligible fashion and written in standard English?

Reviewer #1: No

Reviewer #2: Yes

**********

**Reviewer #1:** ＜Overall assessment＞

Thank you for the opportunity to review this manuscript, which prospectively evaluates postoperative procalcitonin (PCT) levels as a predictor of postoperative complications (POC) after liver resection in a single-center cohort. The topic is clinically relevant, and the use of PCT as an early postoperative biomarker is of interest. However, there are several important issues in study design, statistical analysis, and data presentation that need to be addressed before the manuscript can be considered for publication. I would recommend major revision.

Major comments

① Definition of postoperative complications and mismatch with PCT biology

The primary endpoint “postoperative complications (POC)” appears to include a heterogeneous mixture of infectious and non-infectious events (e.g., PHLF, bile leak, wound complications, etc.), which are analyzed together as a single composite outcome. PCT, however, is primarily a biomarker of bacterial infection rather than non-infectious complications. Using “all POC” as a single endpoint may therefore dilute the true association between PCT and clinically relevant infectious events.

② Inconsistencies in group definitions and implausible results

There seems to be a discrepancy between the description of Group A and Group B in the Methods and the way these groups are used in the Results and Tables. In parts of the text, Group A is described as patients with complications and Group B as those without complications, but elsewhere the opposite appears to be implied. This is compounded by the unexpected finding that the “complication” group has lower intraoperative blood loss and shorter hospital stay, which is counterintuitive.

This raises serious concerns that:

・The labeling of Group A vs Group B may have been inadvertently reversed.

・The statistical analyses may have been performed or interpreted using incorrect group assignments.

The authors should carefully re-check the group definitions, confirm the correct assignment of each patient to each group, and re-run all analyses if necessary. Any corrections must be transparently described in the revised manuscript.

③ Sample size limitations and robustness of ROC analysis

The study includes only 42 patients, with approximately half experiencing complications. Although ROC analysis is performed and a PCT cut-off value is proposed, the small sample size significantly limits the precision and robustness of these estimates.

To improve the credibility of the findings, the authors should:

・Report 95% confidence intervals for AUC, sensitivity, and specificity.

・Clearly state the method of cut-off selection (e.g., Youden index).

・Discuss the risk of overfitting and optimism bias in deriving a cut-off from such a small cohort, and present the conclusions more cautiously.

④ Lack of multivariable analysis and adjustment for confounders

Several clinical variables known to impact postoperative outcomes (e.g., comorbidities such as diabetes, MELD score or liver function indices, extent of resection, concomitant intra-abdominal procedures) appear to differ between groups or could plausibly affect both PCT levels and complication risk.

Without adjusting for these potential confounders, it is difficult to conclude that PCT is an independent predictor of POC.

I recommend:

・Performing a multivariable logistic regression analysis including PCT and key clinical covariates.

・Clearly reporting the adjusted odds ratios, 95% CIs, and p-values.

・If the small sample size limits the number of variables, this limitation should be explicitly acknowledged and the interpretation of PCT as an “independent” marker should be toned down.

⑤ Statistical methods for continuous variables

For variables such as blood loss and length of hospital stay, which are typically skewed, it is unclear whether the assumptions of parametric tests (e.g., t-tests) are met.

The authors should:

・Indicate whether normality was assessed (e.g., using Shapiro–Wilk test or visual inspection).

・Use non-parametric tests (e.g., Mann–Whitney U test) where appropriate.

・Consider presenting continuous variables as median (IQR) rather than mean ± SD if distributions are skewed.

⑥ Positioning within the existing literature and omission of key references

The discussion of prior literature on PCT after hepatectomy is relatively limited. In particular, a recent large-scale study by Mori et al. (Digestive Surgery, 2025; exact citation to be checked by the authors) evaluated postoperative PCT as a predictor of infectious complications in a substantially larger cohort and demonstrated that PCT on POD 1 and POD 3 are independent predictive markers.

This work is highly relevant and should be:

・Cited in the Introduction and/or Discussion, and

・Used to compare and contrast the present findings, especially with respect to:

→The type of endpoint (infectious complications vs all postoperative complications),

→Sample size and statistical power,

→Optimal PCT cut-off values and AUCs,

→Differences in patient population and surgical procedures.

A more thorough discussion of how the current small prospective study adds to or differs from these larger retrospective data would help clarify the incremental value of the present work.

⑦ Generalizability and patient population

The majority of patients in this cohort appear to have benign disease (e.g., hepatolithiasis), and the study was conducted at a single center in Bangladesh. This differs substantially from many hepatectomy series in which malignant liver tumors predominate.

The authors should expand the Discussion to:

・Address how the high proportion of benign disease and unique local disease patterns may influence complication profiles and PCT dynamics.

・Discuss the external validity of their findings and how they might (or might not) generalize to centers treating mainly malignant liver tumors or different patient populations.

Minor comments

① Clarity and consistency of Tables and figure references

・There are several inconsistencies between the numbering of Tables and the references to them in the text.

・The authors should carefully renumber and cross-check all Tables and figures, ensuring that each is called out in a logical order and that the content matches the description in the text.

② Clarification of PCT measurement protocol

・The Methods section should more clearly specify the timing of PCT measurements (exact postoperative days and time points), the assay used, and how values below/above the detection range were handled.

・If any PCT measurements were missing, the handling of missing data should be described.

③ Definition and grading of complications

・It would be helpful to state explicitly whether complications were classified according to a standard system such as the Clavien–Dindo classification, and if so, which grades were included in the definition of POC.

・Presenting a brief summary of the distribution of complication grades would improve interpretability.

④ Language and style

・While the manuscript is generally understandable, there are multiple issues with grammar, tense, and phrasing that may impede readability.

・I recommend that the authors have the manuscript carefully edited by a native English speaker or a professional editing service before resubmission.

⑤ Ethics and data availability

・The ethics approval is mentioned, but it would be useful to include the approval number and date if available.

・Given PLOS ONE’s data availability policy, the authors should ensure that the underlying dataset (appropriately anonymized) is accessible as supplementary material or via a public repository, and that the Data Availability Statement explicitly describes this.

**Reviewer #2:** General Assessment

This manuscript presents a prospective observational study evaluating serum procalcitonin (PCT) as an early predictor of postoperative complications (POC) following elective liver resection. The topic is clinically relevant, particularly in resource-limited settings, and aligns with the scope of PLOS ONE, which emphasizes methodological soundness rather than novelty.

The study demonstrates that postoperative day (POD) 1 PCT is associated with postoperative complications and shows moderate discriminatory ability. However, several methodological and analytical limitations currently limit the strength of the conclusions. In particular, issues related to confounding, group imbalance, outcome definition, and statistical modeling need to be addressed before the manuscript can be considered for publication.

Overall recommendation: Major Revision

Major Comments

1. Confounding and lack of multivariable analysis

The comparison between patients with and without postoperative complications is based exclusively on univariate analyses. However, several baseline and perioperative variables differ significantly between groups, including:

Sex distribution, MELD score, diabetes mellitus, presence of concomitant intra-abdominal procedures

These factors are known to influence postoperative outcomes after liver resection and may independently affect inflammatory markers, including PCT.

Major concern: Without multivariable adjustment (e.g., logistic regression), it is not possible to conclude that POD 1 PCT is an independent predictor of postoperative complications.

2. Definition and heterogeneity of postoperative complications

All postoperative complications—ranging from Clavien-Dindo grade I events to grade B post-hepatectomy liver failure—were grouped together as a single outcome.

Major concern:

This approach introduces substantial clinical heterogeneity. Minor complications (e.g., wound issues) and major liver-specific complications (e.g., PHLF, bile leak) may have very different inflammatory profiles and clinical implications.

Recommendation:

Clearly state the Clavien-Dindo grade distribution of complications.

Perform a sensitivity analysis limited to clinically relevant complications (e.g., ≥ Clavien-Dindo II or III), or justify why inclusion of grade I events is appropriate.

3. Interpretation of ROC analysis and cutoff value

The ROC analysis shows AUC values around 0.79 for POD 1 PCT, indicating moderate discriminatory ability.

Major concern:

The manuscript occasionally implies strong predictive performance, which may overstate the clinical utility of PCT given: The small sample size (n = 42); wide confidence intervals; lack of external validation

Recommendation:

Temper the language regarding “reliable” or “strong” prediction.

Explicitly state that the proposed cutoff (1.100 µg/L) is exploratory and hypothesis-generating, not definitive.

4. Counterintuitive perioperative findings

Patients who developed postoperative complications reportedly had:

Lower intraoperative blood loss

Shorter postoperative hospital stays

These findings contradict expected clinical patterns and are not adequately explained.

Recommendation:

The authors should clarify whether hospital stay refers to total stay or postoperative stay only.

Discuss possible explanations (e.g., early discharge bias, type of complications, healthcare system factors).

Consider whether these findings suggest residual confounding.

5. Lack of preoperative PCT measurements

Preoperative baseline PCT levels were not measured.

Major concern:

Without baseline values, it is unclear whether postoperative PCT elevation reflects surgery-related inflammation, pre-existing conditions, or early infectious complications.

Recommendation:

This limitation should be more prominently discussed, particularly regarding the interpretation of absolute PCT cutoff values.

Minor Comments

1. Clarify group labeling:

There appears to be inconsistency in the manuscript regarding Group A and Group B definitions (complication vs. no complication). Please ensure consistent labeling throughout text, tables, and figures.

2. Statistical assumptions:

Please confirm whether normality assumptions were tested before applying independent sample t-tests, especially given small subgroup sizes.

3. Figures:

Figure legends should explicitly state whether error bars represent SD or 95% CI.

ROC figure (Figure 2D) would benefit from clearer labeling of curves and AUC values.

Language and style:

Minor grammatical and stylistic issues are present and should be addressed during revision. A careful language edit is recommended.

Final Summary Recommendation to the Editor

This manuscript addresses a clinically relevant question and fits within the aims of PLOS ONE. However, due to limitations in statistical analysis, outcome definition, and interpretation of results, major revision is required. Addressing confounding through multivariable analysis and clarifying outcome stratification would substantially strengthen the manuscript.

**********

what does this mean?). If published, this will include your full peer review and any attached files.

If you choose “no”, your identity will remain anonymous but your review may still be made public.

**Do you want your identity to be public for this peer review?**  For information about this choice, including consent withdrawal, please see our Privacy Policy

Reviewer #1: No

Reviewer #2: No

**********

[NOTE: If reviewer comments were submitted as an attachment file, they will be attached to this email and accessible via the submission site. Please log into your account, locate the manuscript record, and check for the action link "View Attachments". If this link does not appear, there are no attachment files.]

To ensure your figures meet our technical requirements, please review our figure guidelines: https://journals.plos.org/plosone/s/figures

You may also use PLOS’s free figure tool, NAAS, to help you prepare publication quality figures: https://journals.plos.org/plosone/s/figures#loc-tools-for-figure-preparation.

NAAS will assess whether your figures meet our technical requirements by comparing each figure against our figure specifications.

# Author comment: Author response to Decision Letter 1

**DOI:** 10.1371/journal.pone.0338171.r002

27 May 2026

We sincerely thank the Academic Editor and Reviewers for their thoughtful and constructive comments. We have carefully revised the manuscript accordingly. All major concerns were addressed through re-analysis of the dataset, revision of statistical methods, clarification of group definitions, addition of exploratory multivariable analyses, sensitivity analyses, updated ROC analyses with confidence intervals, strengthening of the Discussion, and comprehensive language editing. Revised sections are highlighted in the manuscript. An attachment of a point by point response to reviewer attached .

Attachment Submitted filename: Author_response.docx

# Peer review: Decision Letter 1

**DOI:** 10.1371/journal.pone.0338171.r003

**Authors:** Taiichi Wakiya

8 Jul 2026

Serum Procalcitonin as an Early Marker Associated with   Postoperative Complications Following Liver Resection: A Prospective Observational Study

PLOS One

Dear Dr. Islam,

Thank you for submitting your manuscript to PLOS ONE. After careful consideration, we feel that it has merit but does not fully meet PLOS ONE’s publication criteria as it currently stands. Therefore, we invite you to submit a revised version of the manuscript that addresses the points raised during the review process.

Please submit your revised manuscript by Aug 22 2026 11:59PM. If you will need more time than this to complete your revisions, please reply to this message or contact the journal office at plosone@plos.org. When you're ready to submit your revision, log on to https://www.editorialmanager.com/pone/ and select the 'Submissions Needing Revision' folder to locate your manuscript file.

- A letter that responds to each point raised by the academic editor and reviewer(s). You should upload this letter as a separate file labeled 'Response to Reviewers'.
- A marked-up copy of your manuscript that highlights changes made to the original version. You should upload this as a separate file labeled 'Revised Manuscript with Track Changes'.
- An unmarked version of your revised paper without tracked changes. You should upload this as a separate file labeled 'Manuscript'.

If you would like to make changes to your financial disclosure, please include your updated statement in your cover letter. Guidelines for resubmitting your figure files are available below the reviewer comments at the end of this letter.

If applicable, we recommend that you deposit your laboratory protocols in protocols.io to enhance the reproducibility of your results. Protocols.io assigns your protocol its own identifier (DOI) so that it can be cited independently in the future. For instructions see: https://journals.plos.org/plosone/s/submission-guidelines#loc-laboratory-protocols. Additionally, PLOS ONE offers an option for publishing peer-reviewed Lab Protocol articles, which describe protocols hosted on protocols.io. Read more information on sharing protocols at https://plos.org/protocols?utm_medium=editorial-email&utm_source=authorletters&utm_campaign=protocols.

As the corresponding author, your ORCID iD is verified in the submission system and will appear in the published article. PLOS supports the use of ORCID, and we encourage all coauthors to register for an ORCID iD and use it as well. Please encourage your coauthors to verify their ORCID iD within the submission system before final acceptance, as unverified ORCID iDs will not appear in the published article. *Only*  the individual author can complete the verification step; PLOS staff *cannot*  verify ORCID iDs on behalf of authors.

We look forward to receiving your revised manuscript.

Kind regards,

Taiichi Wakiya

Academic Editor

PLOS One

Journal Requirements:

If the reviewer comments include a recommendation to cite specific previously published works, please review and evaluate these publications to determine whether they are relevant and should be cited. There is no requirement to cite these works unless the editor has indicated otherwise.

[Note: HTML markup is below. Please do not edit.]

Reviewers' comments:

Reviewer's Responses to Questions

**Comments to the Author**

Reviewer #1: (No Response)

**********

2. Is the manuscript technically sound, and do the data support the conclusions?

Reviewer #1: Yes

**********

3. Has the statistical analysis been performed appropriately and rigorously?

Reviewer #1: Yes

**********

4. Have the authors made all data underlying the findings in their manuscript fully available?

The PLOS Data policy

Reviewer #1: Yes

**********

5. Is the manuscript presented in an intelligible fashion and written in standard English?

Reviewer #1: Yes

**********

Reviewer #1: The authors have revised the manuscript substantially, and the study addresses a clinically relevant issue: early identification of postoperative complications after liver resection, particularly in a resource-limited setting. The prospective design and serial measurement of PCT, CRP, and WBC are strengths. The addition of ROC analyses, confidence intervals, and exploratory multivariable analysis has improved the manuscript.

However, several issues still need to be addressed before publication.

・The main endpoint is a heterogeneous composite of postoperative complications, including infectious and non-infectious events. Since procalcitonin is biologically more closely related to infection and systemic inflammatory response, the authors should be more cautious in interpreting PCT as a predictor of overall postoperative morbidity. The conclusion should consistently state that POD1 PCT was “associated with” postoperative complications and showed “moderate discriminatory ability,” rather than implying that it is a reliable or specific predictor of all complications.

・The sample size is small, with only 42 patients and 22 events. The multivariable model includes several covariates despite the limited number of events, and the confidence intervals are very wide. Therefore, the adjusted odds ratios should be interpreted as exploratory. I recommend further softening the wording around “independent predictor” throughout the Abstract, Results, and Discussion.

・The sensitivity analyses are important and should be discussed more clearly. The AUC decreased for clinically relevant complications, especially for Clavien–Dindo grade ≥III complications. This suggests that the clinical utility of POD1 PCT for predicting severe morbidity is limited in the present dataset. This point should be acknowledged more explicitly.

・The authors cite previous studies on PCT after hepatectomy, including the recent study by Mori et al. However, the distinction between infection-specific endpoints in previous studies and the broad composite endpoint used in the present study should be emphasized more clearly. This would help readers understand the novelty and limitations of the current work.

・There are still some issues with presentation. The figure numbering appears confusing in several places, and some descriptions of statistical methods are inconsistent, for example the use of median/IQR together with wording suggesting a t-test. The authors should carefully check all tables, figure legends, and statistical footnotes.

Overall, I think the manuscript may be suitable for PLOS ONE if the conclusions are further tempered and the limitations of the small sample size, heterogeneous endpoint, and exploratory analyses are clearly stated.

**********

what does this mean?). If published, this will include your full peer review and any attached files.

If you choose “no”, your identity will remain anonymous but your review may still be made public.

**Do you want your identity to be public for this peer review?**  For information about this choice, including consent withdrawal, please see our Privacy Policy

Reviewer #1: No

**********

[NOTE: If reviewer comments were submitted as an attachment file, they will be attached to this email and accessible via the submission site. Please log into your account, locate the manuscript record, and check for the action link "View Attachments". If this link does not appear, there are no attachment files.]

To ensure your figures meet our technical requirements, please review our figure guidelines: https://journals.plos.org/plosone/s/figures

You may also use PLOS’s free figure tool, NAAS, to help you prepare publication quality figures: https://journals.plos.org/plosone/s/figures#loc-tools-for-figure-preparation.

NAAS will assess whether your figures meet our technical requirements by comparing each figure against our figure specifications.

# Author comment: Author response to Decision Letter 2

**DOI:** 10.1371/journal.pone.0338171.r004

19 Aug 2026

Response to Reviewer

Manuscript ID: PONE-D-25-62019R1

Title: Serum Procalcitonin as an Early Marker Associated with Postoperative Complications Following Liver Resection: A Prospective Observational Study

Dear Academic Editor and Reviewer,

We sincerely thank you for the careful re-review and constructive comments. We have revised the manuscript to temper causal/predictive language, clarify the exploratory nature of the adjusted analyses, explicitly discuss the reduced discrimination for clinically relevant and severe complications, distinguish our broad composite endpoint from infection-specific endpoints in prior studies, and correct figure/statistical presentation inconsistencies. All changes are highlighted in tracked change manuscript.

Comment 1: Heterogeneous composite endpoint and overly strong interpretation of PCT.

Response: We agree. We have consistently reframed POD1 PCT as being “associated with” postoperative complications and as showing “moderate discriminatory ability,” rather than as a reliable or specific predictor of all postoperative morbidity. We also explicitly state that the composite endpoint includes infectious and non-infectious events and that PCT may reflect infection, tissue injury, and postoperative inflammatory physiology.

Changes: Abstract; Discussion paragraphs addressing interpretation and biological specificity; Conclusion.

Comment 2: Small sample size, limited events, wide confidence intervals, and wording around “independent predictor.”

Response: We agree. We now explicitly describe the multivariable analysis as exploratory, note the low number of events and very wide confidence intervals, and avoid interpreting the adjusted estimates as definitive evidence of independent prediction. The Results, Discussion, limitations, and Conclusion have been softened accordingly.

Changes: Abstract; Results paragraph accompanying Table 6; Discussion; Limitations; Conclusion.

Comment 3: Sensitivity analyses and limited utility for severe morbidity should be discussed more clearly.

Response: We agree. We now emphasize that discrimination decreased when stricter clinically relevant endpoints were used: AUC 0.650 for Clavien–Dindo grade ≥II and 0.581 for grade ≥III complications. We explicitly acknowledge that the low AUC for grade ≥III morbidity indicates limited clinical discrimination for severe complications in this dataset.

Changes: Abstract; Results; Discussion; Limitations; Conclusion.

Comment 4: Clarify distinction between infection-specific endpoints in previous studies and the broad composite endpoint in the present study.

Response: We agree. We revised the comparison with Li et al., Mori et al., and Aoki et al. to make clear that previous studies often used infection-specific or clinically restricted endpoints, whereas our primary endpoint included both infectious and non-infectious complications. We now present this difference as both a conceptual distinction and a limitation of our study.

Changes: Discussion paragraphs comparing the present findings with Li et al., Mori et al., and Aoki et al.

Comment 5: Figure numbering and inconsistent statistical descriptions.

Response: We carefully reviewed the relevant presentation. The text describing serial WBC, CRP, and PCT now refers to Figure 2A–C, while the ROC analysis refers to Figure 2D; Figure 1 remains the distribution of postoperative complications. We also corrected the Figure 2 legend from “mean” to “median” for the skewed biomarkers and removed the contradictory Table 4 footnote stating that an independent-samples t-test was used. Table 4 now states that the data are median (IQR) and were compared using Mann–Whitney U tests, consistent with the Statistical Analysis section.

Changes: Results cross-references; Figure 2 legend; Table 4 footnote; Table 5 title.

We appreciate the reviewer’s comments, which have helped us improve the precision and clarity of the manuscript.

Sincerely,

The Authors

Attachment Submitted filename: Response_to_Reviewer.docx

# Peer review: Decision Letter 2

**DOI:** 10.1371/journal.pone.0338171.r005

**Authors:** Taiichi Wakiya

23 Aug 2026

Serum Procalcitonin as an Early Marker Associated with Postoperative Complications Following Liver Resection: A Prospective Observational Study

PONE-D-25-62019R2

Dear Dr. Islam,

We’re pleased to inform you that your manuscript has been judged scientifically suitable for publication and will be formally accepted for publication once it meets all outstanding technical requirements.

Within one week, you’ll receive an e-mail detailing the required amendments. When these have been addressed, you’ll receive a formal acceptance letter and your manuscript will be scheduled for publication.

An invoice will be generated when your article is formally accepted. Please note, if your institution has a publishing partnership with PLOS and your article meets the relevant criteria, all or part of your publication costs will be covered. Please make sure your user information is up-to-date by logging into Editorial Manager at Editorial Manager® and clicking the ‘Update My Information' link at the top of the page. For questions related to billing, please contact billing support.

If your institution or institutions have a press office, please notify them about your upcoming paper to help maximize its impact. If they’ll be preparing press materials, please inform our press team as soon as possible -- no later than 48 hours after receiving the formal acceptance. Your manuscript will remain under strict press embargo until 2 pm Eastern Time on the date of publication. For more information, please contact onepress@plos.org.

Kind regards,

Taiichi Wakiya

Academic Editor

PLOS One

Additional Editor Comments (optional):

Reviewers' comments:

Reviewer's Responses to Questions

**Comments to the Author**

Reviewer #1: All comments have been addressed

**********

2. Is the manuscript technically sound, and do the data support the conclusions?

Reviewer #1: Yes

**********

3. Has the statistical analysis been performed appropriately and rigorously?

Reviewer #1: I Don't Know

**********

4. Have the authors made all data underlying the findings in their manuscript fully available?

The PLOS Data policy

Reviewer #1: Yes

**********

5. Is the manuscript presented in an intelligible fashion and written in standard English?

Reviewer #1: Yes

**********

Reviewer #1: The authors have adequately addressed my previous comments, and I consider the revised manuscript acceptable for publication.

**********

what does this mean?). If published, this will include your full peer review and any attached files.

If you choose “no”, your identity will remain anonymous but your review may still be made public.

**Do you want your identity to be public for this peer review?**  For information about this choice, including consent withdrawal, please see our Privacy Policy

Reviewer #1: No

**********

# Editor report: Acceptance letter

**DOI:** 10.1371/journal.pone.0338171.r006

**Authors:** Taiichi Wakiya

PONE-D-25-62019R2

PLOS One

Dear Dr. Islam,

I'm pleased to inform you that your manuscript has been deemed suitable for publication in PLOS One. Congratulations! Your manuscript is now being handed over to our production team.

At this stage, our production department will prepare your paper for publication. This includes ensuring the following:

* All references, tables, and figures are properly cited

* All relevant supporting information is included in the manuscript submission,

* There are no issues that prevent the paper from being properly typeset

You will receive further instructions from the production team, including instructions on how to review your proof when it is ready. Please keep in mind that we are working through a large volume of accepted articles, so please give us a few days to review your paper and let you know the next and final steps.

Lastly, if your institution or institutions have a press office, please let them know about your upcoming paper now to help maximize its impact. If they'll be preparing press materials, please inform our press team within the next 48 hours. Your manuscript will remain under strict press embargo until 2 pm Eastern Time on the date of publication. For more information, please contact onepress@plos.org.

You will receive an invoice from PLOS for your publication fee after your manuscript has reached the completed accept phase. If you receive an email requesting payment before acceptance or for any other service, this may be a phishing scheme. Learn how to identify phishing emails and protect your accounts at https://explore.plos.org/phishing.

If we can help with anything else, please email us at customercare@plos.org.

Thank you for submitting your work to PLOS One and supporting open access.

Kind regards,

PLOS One Editorial Office Staff

on behalf of

Dr. Taiichi Wakiya

Academic Editor

PLOS One
